# 亚实性肺结节CT三维质量分析：观察者内及观察者间差异

**DOI:** 10.3779/j.issn.1009-3419.2015.05.06

**Published:** 2015-05-20

**Authors:** 慧婷 刘, 颖 王, 磊 冯, 铁链 于

**Affiliations:** 300052 天津，天津医科大学总医院放射科 Department of Radiology, Tianjin Medical University General Hospital, Tianjin 300052, China

**Keywords:** 亚实性结节, 质量测量, 体积测量, 计算机断层扫描, 重复性, Subsolid nodules, Mass measurement, Volume measurement, Computed tomography, Repeatability

## Abstract

**背景与目的:**

肺内亚实性结节（包括磨玻璃密度结节和部分实性结节）体积增长常较缓慢，但恶性概率比实性结节大，常需随访确定其生长特性。恶性亚实性结节的生长性不仅可以表现为体积的增长，也可以表现为密度的增加或新出现实性成分。计算机断层扫描（computed tomography, CT）质量（Mass）测量能综合评估其体积及密度的变化，在结节随访中量化反映其生长特性。本研究目的是评估亚实性结节质量测量的重复性，并与体积测量重复性比较。

**方法:**

两名医生盲法应用结节分析软件对44例患者共80个亚实性结节的CT影像资料进行三维体积及质量重复测量，对自动分割效果不佳的结节采用手动调整。应用Bland-Altman法评估质量测量及体积测量的观察者内及观察者间差异，组内相关及*Wilcoxon*检验评估质量测量与体积测量观察者内、间测量变异度的差异。

**结果:**

74个（92.5%）亚实性结节分割效果满意纳入研究。结节直径（7.2±2.5）mm（3.2 mm-16.4 mm）。质量测量的观察者内、观察者间95%一致性区间分别为-11.5%-10.4%、-17.4%-19.3%，体积测量的观察者内、观察者间95%一致性区间分别为-8.4%-8.8%、-17.9%-19.4%，观察者内、间质量测量与体积测量变异度的组内相关系数分别为0.95、0.93（*P*均 < 0.001），二者之间无统计学差异（*P*值分别为0.78、0.09）。手动调整结节分割对测量的重复性有一定影响。

**结论:**

亚实性肺结节的质量测量重复性较好，可作为随访定量评估方法。

## Abstract (en)

**Background and objective:**

Subsolid pulmonary nodules tend to exhibit considerably slower growth rates than solid lesions, nevertheless, higher malignancy probability. The diagnosis of indeterminate nodules largely depends on the growth evaluation during follow-up. The growth can manifested as an increase in size or the appearance and/or subsequent increase of solid components. The mass reflect the product of volume and density and can be more sensitive in growth evaluation. However, the repeatability needs a further validation. The purpose of this study is to assess the intra and inter-observer variability of mass measurement for subsolid nodules.

**Methods:**

80 subsolid nodules in 44 patients were retrospectively enrolled. Both the volume and mass were measured by two radiologists using blind method independently. Intra-observer and inter-observer variability were analyzed and compared by Bland-Altman method intra-class correlation test and Wilcoxon test.

**Results:**

Software achieved satisfied segmentation for 92.5% nodules. Of them, 35% underwent manual modification. The 95% limits of agreement for intra-observer variability were -11.5%-10.4% for mass and -8.4%-8.8% for volume. The 95% limits of agreement for inter-observer variability were -17.4%-19.3% for mass and -17.9%-19.4% for volume.The intra-class correlation foefficients between volume and mass measument was 0.95 and 0.93 (both *P* < 0.001) and no significant differences (*P*=0.78, 0.09) was found for intra- and inter-observer variability. Manual modification of the segmentation caused the worse mass measurement repeatability in spite of the reader satisfaction.

**Conclusion:**

The repeatability of mass measurement has no significant difference with that of volume measurement and may act as a reliable method in the follow-up of subsolid nodules.

肺癌的患病率和死亡率在所有恶性肿瘤中目前均居首位^[1]^。腺癌约占全部肺癌的一半，是最常见的肺癌组织学亚型^[2]^。长期持续存在的亚实性结节（包括纯磨玻璃密度结节和部分实性结节）^[3]^与局限性纤维化、不典型腺瘤样增生、原位腺癌、微侵润腺癌、伏壁生长为主的腺癌相关^[4-7]^，影像形态学鉴别困难，正电子发射型计算机断层扫描（positron emission computed tomography, PET）诊断常呈假阴性^[8]^，计算机断层扫描（computed tomography, CT）引导下穿刺活检诊断率低^[9]^。目前对于定性困难的亚实性结节，临床常通过随访测量其体积变化来确定其生长性，并以此作为鉴别诊断的主要依据。但是，恶性亚实性结节的体积生长常呈惰性^[10]^；其生长性不仅可以表现为体积的增长，也可以表现为密度的增加或新出现实性成分^[11]^。为此，最近国外研究者提出质量（Mass）的概念^[11]^，认为质量测量能更敏感地监测出亚实性结节的生长变化。但目前对亚实性结节质量测量有效性的重要依据——测量的可重复性的证据尚少，国内尚未见报道。因此，本研究目的是，评估亚实性结节质量测量的可重复性，并比较亚实性结节质量及体积测量在观察者内及观察者间的差异。

## 1 对象和方法

### 1.1 患者资料

回顾性分析2013年12月-2014年10月间天津医科大学总医院放射科行CT检查发现亚实性结节并在我院进行随访的患者。入选条件：持续存在3个月以上的亚实性结节，结节大小3 mm-2 cm。共有44例符合条件的患者（男性20例），年龄范围40岁-85岁，平均年龄（62±10.3）岁，符合条件的结节共80个（表 1）。

**1:** 亚实性结节的特性 Characteristic of nodule in the study

Characteristic		*n*	Proportion
Density	Part-solid	21	26.25%
	Pure GGO	59	73.75%
Diameter	3 mm-5 mm	13	16.25%
	5 mm-10 mm	58	72.50%
	10 mm-15 mm	8	10.00%
	15 mm-20 mm	1	1.25%
Location	Vascular -attached	34	42.50%
	Pleural-attached	18	22.50%
	In the pulmonary parenchyma	28	35.00%
GGO: ground-glass opacity.			

### 1.2 CT图像采集

所有检查均使用64排螺旋CT机（GE Light Speed）进行，扫描范围自胸廓入口至肺底部。患者一次吸气后屏气完成全肺扫描，螺旋扫描方式，电压120 KV，电流300 mA，螺距1.375:1，层厚5 mm，机架旋转一周时间0.4 s，显示野（field of view, FOV）360 mm，图像矩阵512×512，默认重建算法为标准算法，重建1.25 mm层厚轴位图像。

### 1.3 亚实性结节体积与质量测量

亚实性结节体积与质量测量均在GE AW4.6工作站进行。由二位放射科医生分别独立测量并由其中一位间隔一周重复测量。应用高级肺结节分析软件（advanced lung analysis, ALA），具体操作步骤：选择要进行测量的一组轴位图像（1.25 mm）数据，进入容积分析界面后，在打开的轴位图像上由观察者用鼠标单击目标结节，再选择亚实性结节的位置选项（肺实质内、毗邻血管或胸膜），而后由软件根据所选结节的位置选择相应的算法对结节进行分割并显示出亚实性结节的分割图像。操作者在轴、矢及冠状位图像上观察结节分割情况，对分割不满意的亚实性结节可以通过调整鼠标的选取点、设定结节周围提取范围（3 mm-30 mm）进行调整以达到最佳效果，如果经调整后结节分割的区域刚好完整的与结节重合，则结果为分割满意（图 1，图 2），否则确定为分割不满意并从研究对象中剔除。软件自动计算出分割后结节的体积V（mm^3^）及平均CT值（HU）。根据CT值与物理密度的线性相关性^[12, 13]^，将测出的平均CT值+1, 000转换为假定物理密度ρ（单位为mg/cm^3^）。采用公式m=ρ*V/1, 000计算出亚实性结节的质量m（单位为mg）^[14]^。

**1:**
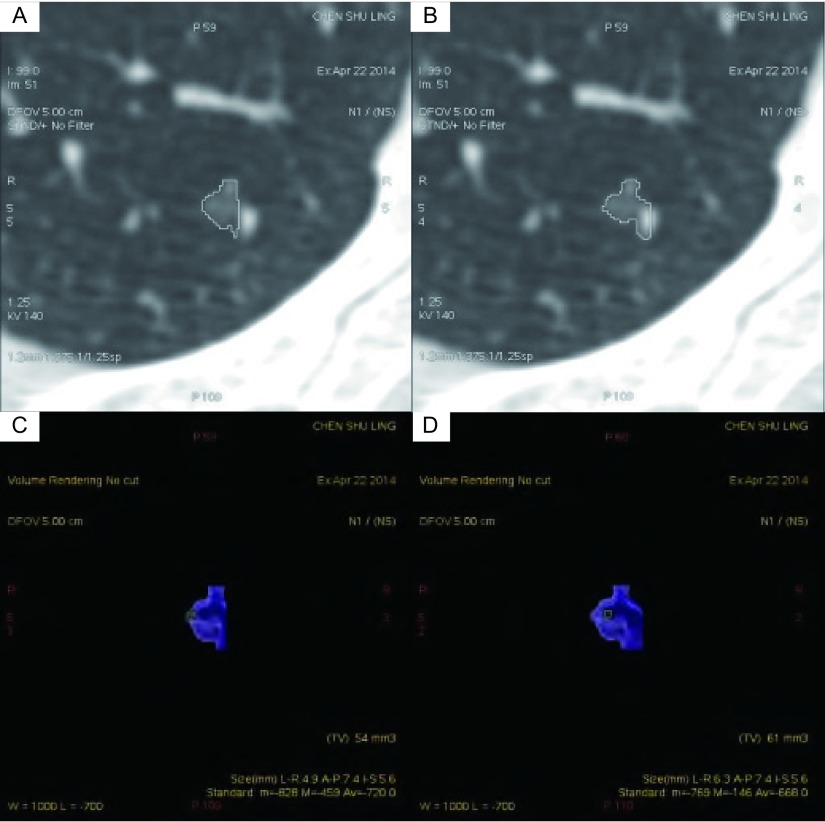
女，55岁，右下叶磨玻璃密度结节，直径约为6.0 mm。图A、C为观察者1第一次测量，大小为54 mm^3^，密度为280 mg/cm^3^，质量约为15.120 mg。图B、D为观察者1第二次测量，大小为61 mm^3^，密度为332 mg/cm^3^，质量约为20.252 mg。质量变异程度与体积变异程度分别为29.0%、12.2% Volume and attenuation measurement of GGN in a 55-year-old woman by using semiautomatic software program. Thin-section chest CT image in lung window setting shows 6-mm GGN in right lower lobe. A, C: the 1^st^ measurement of observer 1, the size of the GGN is 54 mm^3^ and the atteunuation is -720 HU, so the mass is about 15.120 mg; B, D: the 2^nd^ measurement of observer 1, the size of the GGN is 61 mm^3^ and the atteunuation is -668 HU, so the mass is about 20.252 mg. The variation of the mass and the volume is 29.0%, 12.2%. CT: computed tomography

**2:**
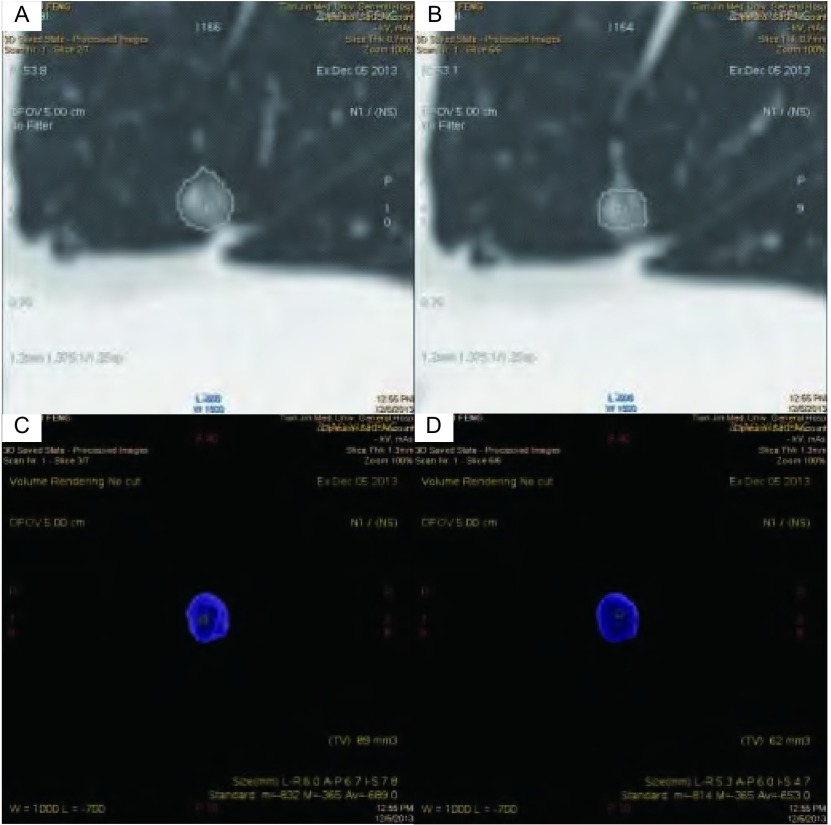
女，53岁，右中叶磨玻璃密度结节，直径约为4.6 mm。图A、C为观察者1第一次测量，大小为89 mm^3^，密度为311 mg/cm^3^，质量约为27.679 mg。图B、D为观察者1第二次测量，大小为62 mm^3^，密度为347 mg/cm^3^，质量约为21.574 mg。质量变异程度与体积变异程度分别为25.0%、35.8% Volume and attenuation measurement of GGN in a 53-year-old woman by using semiautomatic software program. Thin-section chest CT image in lung window setting shows 4.6-mm GGN in right middle lobe. A, C: the 1st measurement of observer 1, the size of the GGN is 89 mm^3^ and the atteunuation is -689 HU, so the mass is about 27.679 mg; B, D: the 2nd measurement of observer 1, the size of the GGN is 62 mm^3^ and the atteunuation is -653 HU, so the mass is about 21.574 mg. The variation of the mass and the volume is 25.0%, 35.8%

### 1.4 统计学方法

应用Bland-Altman法评估体积测量和质量测量观察者内的可重复性，即计算同一医生二次重复测量的平均值（average, Av）及相对差值（relative difference, RD）：RD体积=(V1-V2)/Av，式中V1、V2分别为两次体积测量值（mm^3^）；RD质量=（m1-m2）/Av，式中m1、m2分别为两次质量测量值（mg）。分别取RD的95%置信区间代表观察者内变异度。同样的方法计算两位医生重复测量结果的Av、RD，并以RD的95%CI代表观察者间变异度。以RD做为评价指标，应用组内相关系数（intra-class correlation coefficient, ICC）评价观察者内及观察者间质量测量变异度与体积测量变异度的一致性，*Wilcoxon*配对检验评估质量测量与体积测量RD之间有无明显差异性。所有统计分析都在统计学软件SPSS 17.0中完成，以*P* < 0.05为差异有统计学意义。

## 2 结果

### 2.1 亚实性结节的软件分割效果

80例肺结节的软件分割满意率为92.5%（74/80）；其中58%（46/80）为软件自动分割满意，35%（28/80）经手动调整后分割效果满意，共74个结节纳入研究（表 2）。

**2:** 结节分割及观察者内、观察者间95%一致性区间 The segmentaion of subsolid nodules and the intra- and inter-observer variability

Nodule segmentation	*n*	95%limits of agreements			
		Intra-observer (Volume)	Inter-observer (Volume)	Intra-observer (Mass)	Inter-observer (Mass)
^*^Automatic	46	-8.0%-8.0%	-7.8%-10.0%	-8.2%-7.1%	-7.5%-11.0%
^*^Semi-Auto	28	-23.3%-19.2%	-22.7%-19.5%	-28.1%-28.6%	-27.8%-27.1%
Total	74	-8.4%-8.8%	-17.9%-19.4%	-11.5%-10.4%	-17.4%-19.3%
^*^Automatic: Successful automatic segmentation; ^*^Semi-Auto: Successful segmentation after manual adjustation.					

### 2.2 质量测量与体积测量的重复性

观察者内亚实性结节3D体积测量RD均值为0.2%，95%一致性区间为-8.4%-8.8%；质量测量RD均值为-0.5%，95%一致性区间为-11.5%-10.4%（图 3）。观察者间体积测量RD均值为0.8%，95%一致性区间为-17.9% -19.4%；质量测量RD均值为0.9%，95%一致性区间为-17.4%-19.3%（图 4）。观察者内质量测量RD与体积测量RD的组内相关系数为0.95（*P* < 0.001），观察者间质量测量RD与体积测量RD的组内相关系数为0.93（*P* < 0.001）。体积测量相对差值与质量测量RD之间在观察者内、观察者间均无统计学差异（Z=-0.277, -1.671, *P*=0.09, 0.78; *Wilcoxon* test）。软件自动分割效果满意的结节其质量、体积测量的95%一致性区间均较经手动调整分割的结节范围减小（表 2）。

**3:**
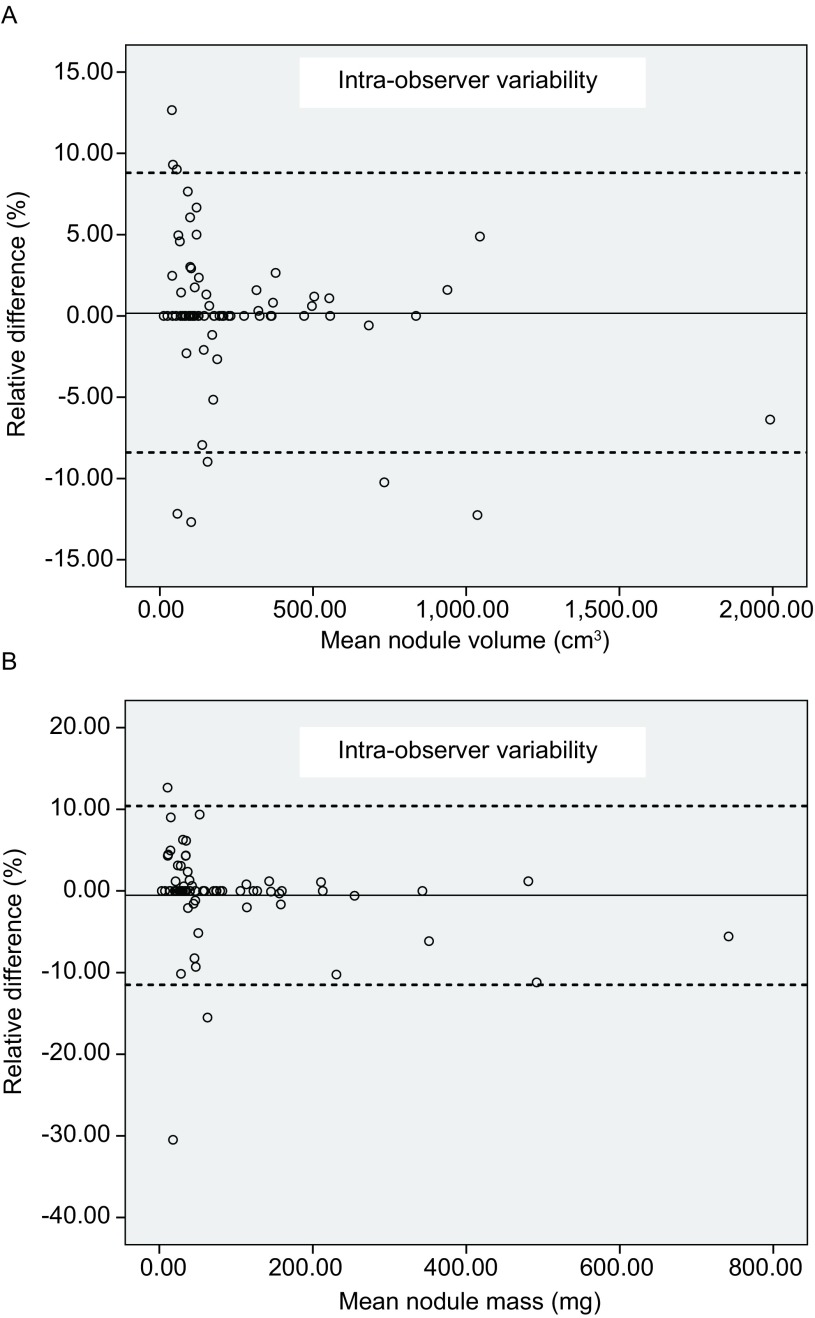
Bland-Altman分布图：图A为观察者内体积变异分布图，图B为观察者内质量变异分布图。实线代表均值，虚线代表 95%一致性区间的上限及下限 Bland-Altman plots. A: The Bland-Altman plots of intra-observer volume measurement variability; B: The Bland-Altman plots of mass measurement variability. Bland-Altman: The dash line represents the mean relative difference, the dot line represents the upper and lower value of 95% limits of agreement

**4:**
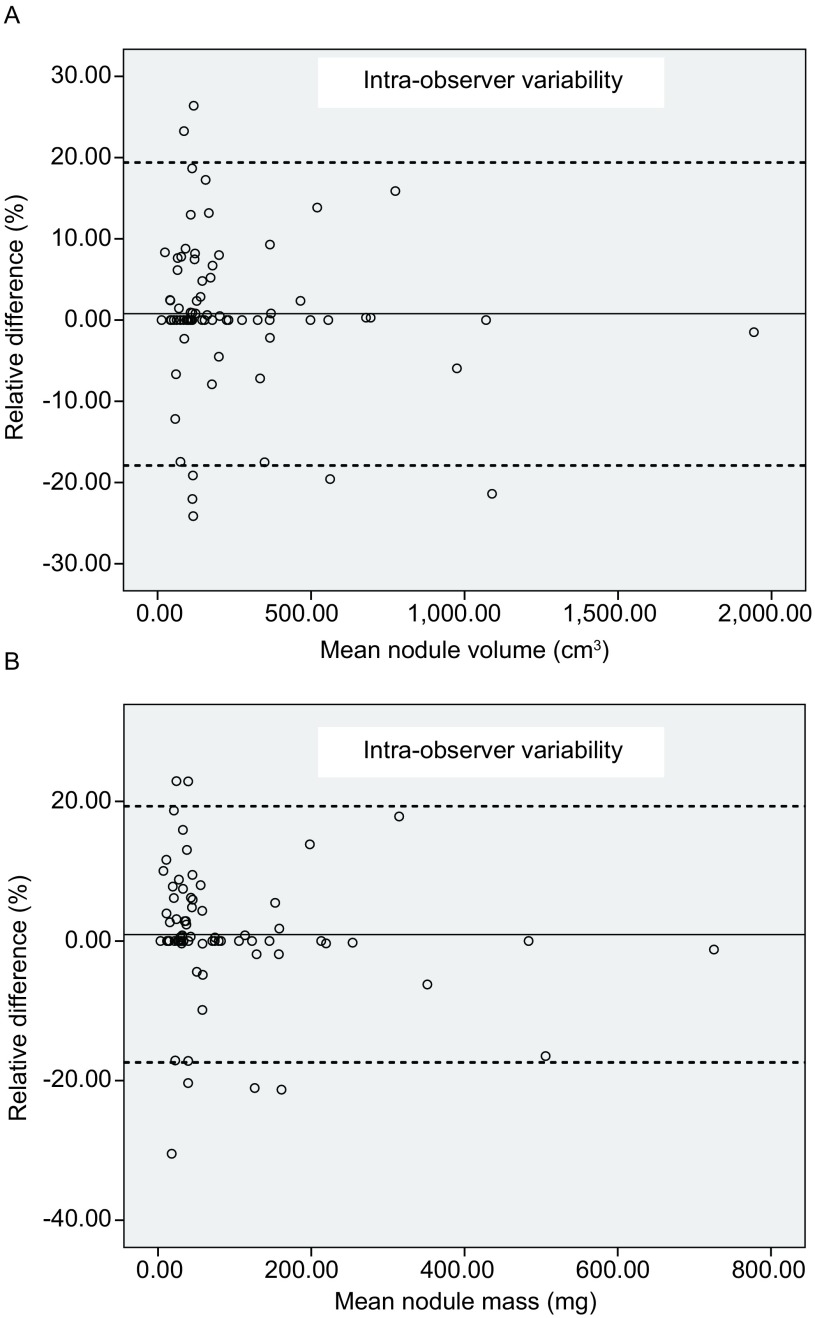
Bland-Altman分布图：图A为观察者间体积变异分布图，图B为观察者间质量变异分布图。实线代表均值，虚线代表 95%一致性区间的上限及下限 Bland-Altman plots. A: The Bland-Altman plots of inter-observer volume measurement variability; B: The Bland-Altman plots of mass measurement variability. Bland-Altman: The dash line represents the mean relative difference, the dot line represents the upper and lower value of 95% limits of agreement

## 3 讨论

肺内亚实性结节体积增长常较缓慢，但恶性概率比实性结节大，常需随访确定其生长特性。如何早期精确确定肺内不定性亚实性结节的生长特性对于肺癌的早诊具有重要的意义。恶性亚实性结节的生长不仅包括体积的增长，还包括密度的增高和实性成分的增加，因此质量（Mass）测量能够综合反映二者的变化，从理论上说可以更准确监测出其生长性。我们的研究证明质量测量具有很好的重复性，而结节的分割方式（Automatic或Semi-Auto）对质量测量的变异度有一定影响。

在本研究中，亚实性结节质量测量观察者内一致性区间为-11.5%-10.4%，观察者间一致性区间为-17.4%-19.3%。这与Kim等^[14]^的近期研究结果相近，他们的结果显示观察者内质量测量的一致性区间为-8.4%-9.4%，观察者间一致性区间为-17.5%-11.8%。质量测量的一致性区间对判定生长性具有重要的意义，区间的上限可设定为判断亚实性结节是否生长的界限。本研究中观察者间的一致性区间上限达19.3%，表明在日常随访中如果质量测量变化率小于19.3%，此差异则有可能由测量的误差引起，无法判定结节是否真正生长；而大于此界限，则可认为结节的质量变化是由于结节的生长引起。

我们发现质量测量与体积测量无论在观察者内还是在观察者间的变异度均存在明显相关，二者之间没有明显差异性，与近期Kim等^[14]^的研究结果相一致。而De Hoop等^[11]^利用人工测量体积的方法显示质量测量与体积测量在变异度上具有明显差异性。其原因可能在于他们采用的测量方法与我们的不一致，我们研究中结节的CT值由软件提供，而De Hoop的研究应用人工测量，人工CT值测量时感兴趣区一般设定在结节中心区域，但亚实性结节并不都是均质的，因此测量的CT值也会随着感兴趣区的位置及大小的不同而变化。本研究中，感兴趣区由软件自动获取，对于分割不满意的结节，我们通过观察不同方位截面的结节分割图像，分析其误差的原因，并根据结节的位置、密度和通过采用不同的算法、调整感兴趣区大小等方法获得满意的分割。因此，其质量的变异度同体积的变异度无明显差异。

结节质量测量结果的重要影响因素之一是结节的分割情况。在本研究中，我们观察到质量变异与体积变异的趋势在少数结节中并不一致，可分为二种情况：①质量变异程度大于体积变异程度（图 1），其原因可能为结节与血管相贴，结节两次分割的差异在于是否包含血管，当包含血管时，血管会造成结节体积的增加，平均密度也增加，而由于血管的密度相对较高，因此质量增加的程度比体积增加的程度大；②质量变异程度小于体积变异程度，其原因可能是两次测量分割的差异发生在结节的外周区（图 2），亚实性结节的外周区多呈浅淡的磨玻璃密度，其密度很低，此部分体积在结节总体积中占比较高，但其质量在总体质量中占比很小，因此，当外周区分割发生变化时，体积变异大而质量变异小。综合上述情况，在日常测量中，重要的是应对亚实性结节进行仔细、准确的分割，以尽可能地减少测量误差。

我们的研究存在一定局限性。首先，我们的测量仅采用的是同一次CT扫描数据。进一步的研究还需要证实患者的体位、呼吸、心脏搏动等因素对测量重复性的影响。其次，本次研究样本数据量较小，还需进一步扩大样本以得出更具总体代表性的结果。

总之，亚实性肺结节的质量测量具有和体积测量相近的较好重复性，可以作为亚实性结节随访的评估方式。
